# An Essay on the Theory and Practice of Medical Electricity

**Published:** 1781-04

**Authors:** 


[ *To be continued.* ]

---

IV. *An Essay on the Theory and Practice of Medical Electricity. By* Tiberius Cavallo, *F. R. S.* 8vo. London, 1780. 112 *pages, with a copper-plate.*

THE medical application of electricity, as it was practised till very lately, and as it is still used by many practitioners, was attended with two inconveniences; the first was the administration of strong shocks, which terrified many a patient, and the second was a long continuance of the application, which was tiresome both for the patient and practitioner. But it has lately been observed, that strong shocks and strong sparks are not only useless, but even hurtful in a variety of cases; and that so far from a long continuance of electrification being necessary, the application of that power for three or four minutes a day, is in general sufficient.

As

As this improved method of applying electricity is at prefent confined to a few practitioners, Mr. Cavallo was induced to compile the effay before us, in order to make it generally known.

The work is divided into three parts. The firft of thefe is allotted *to the theory of medical electricity*, and is prefaced by a concife view of its hiftory. Our author very properly obferves, that a better acquaintance with electricity than philofophers had about thirty or forty years ago, and a lefs faith in the accounts of the generality of thofe perfons, whofe intereft it is to promote the ufe of electricity in phyfic, has pointed out the real effects of that power upon the human body, in various circumftances, and has fhewn how far we may confide in it; eftablifhing upon indifputable facts, that electricity is neither that admirable panacea, as it was confidered by fome fanatical and interefted perfons, nor fo ufelefs an application as others have afferted; but that when properly managed, it is an harmlefs remedy, which is fometimes of fingular efficacy.

Mr. Lovet is fpoken of by our author as the firft writer who contended that the fhocks to be ufed in medical electricity fhould be very fmall. Before the publication of his effay, entitled *Subtil Medium proved*, ftrong fhocks, or at leaft very pungent pungent ſparks, were thought neceſſary to ſti-mulate any diſeaſed part of the human body; but at preſent it is very reaſonably eſtabliſhed upon experience, that the greateſt electric powers which can be applied with good expectations, are exceedingly ſmall ſhocks and moderate ſparks, adminiſtered by means of a wooden point, as it is commonly called, or merely by a metallic point, ſo that the perſon electrified feels only a gentle wind upon that part of the body towards which the point is directed.

It has been confidently affirmed by ſome philoſophers, that electrization increaſes the number of pulſations about one-ſixth; ſo that if the pulſe of a perſon naturally beats eighty times in a minute, it will, after being electrized for a few minutes, beat about ninety-four times in the ſame ſpace of time. Others have ſaid that the increaſe of pulſations is more than a ſixth; and ſome perſons have even aſſerted that the pulſe is not at all increaſed. In the Memoirs of the Academy at Berlin, for 1772, M. Gerhard obſerves, that electrization ſometimes quickens the pulſe ſo much as to double the number of pulſations; and ſometimes retards it conſiderably. It has alſo been aſſerted, that poſitive electricity accelerates the pulſe; and that on the concontrary, negative electricity retards it. But experience authorizes our author to ſay, that this effect varies conſiderably, according to the degree of electrization, and principally according to the natural diſpoſition of the perſon tried, and the degree of apprehenſion with which he ſubjects himſelf to be electrified; but that in general, either poſitive or negative electrization, increaſes the number of pulſations about one-ſixth. He ſuppoſes that the effects uſually obſerved upon the body when electrized, are owing to the irritation or dilatation occaſioned by the action of the electric fluid.

In the ſecond part of his work, Mr. Cavallo gives directions for the practical application of electricity for the cure of various diſeaſes. For this purpoſe he recommends large machines, and for this reaſon, that when the ſtream is uſed, which has lately been found to be more efficacious than ſhocks, then a ſmall machine will be altogether uſeleſs. He obſerves, that the largeſt machines will not be found to afford a ſtream too ſtrong for medical purpoſes; but that the uſeful ones, which do not require a great labour to be put in motion, and may furniſh a ſufficiently denſe ſtream, ſhould have the glaſs globe, or cylinder, at leaſt nine inches in diameter, which, with a proportionate conductor, may usually give sparks about three inches long.

Our author considers it as immaterial with respect to medical electricity, whether the rubber of these machines stands upon a glass pillar or not.

He observes, that the power of electricity should be so regulated, as to apply every degree of it with facility and readiness; beginning with a stream issuing out of a metal point, next using a wooden point, then small sparks, stronger sparks, and lastly, small shocks.

Our author remarks that it is impossible to prescribe the exact degree of electrization that must be used for various disorders; persons of different constitutions, though afflicted with the very same disease, requiring different degrees of electrization. Some are so delicate and irritable, that the smallest sparks give them as much pain as shocks do to others; while, on the contrary, some people can suffer pretty severe shocks without positive pain; and Mr. Cavallo has heard of persons who were insensible of any electric power even from shocks of considerable strength.

He observes, that the operator should always use the smallest degree of electric power that is sufficient for the purpose; and the degree of elecelectrization adminiftered fhould never exceed that which the patient can conveniently fuffer, experience fhewing, that when the application of any degree of electricity is very difagreeable to the patients, they very feldom mend.

We are told that the inftruments, which befides the electrical machine and its prime conductor, are neceffary for the adminiftration of medical electricity, may be reduced to three; viz. an electric jar, with Mr. Lane's electrometer; an infulated chair, or an infulated ftool, upon which a common chair may be occafionally fet, and the *directors*. Each of the latter of thefe inftruments confifts of a knobbed brafs wire, cemented to a glafs handle by means of a brafs cap. The manner of ufing them is by taking hold of the extremities of their brafs handles, and bringing their balls into contact with the extremities of that part of the body of the patient, through which we defire to fend the fhock. When more proper inftruments cannot be had, directors may be made by fticking large pins upon fticks of fealing-wax. The furface of the jar, which is coated with tinfoil, is directed to be about four inches in diameter, and fix inches high, which is equal to about feventy-three fquare inches.

Mr. Cavallo obferves, that when fhocks are adminiftered, it is immaterial whether the patient ftands upon the ground, upon the infulating ftool, or in any other fituation whatever, and that it is not always neceffary to remove the cloaths from the part that is to be electrified; for unlefs they are too many and too thick, the fhocks will go through them very eafily, efpecially if the knobs of the directors be preffed a little upon the part.

Befides the directors juft now mentioned, there is another kind defcribed by our author which differs from the former one in having its wire bent, and in terminating in a point, to which is affixed a piece of wood about an inch, or an inch and a half long, pointed at one end, though not very fharp, and having a hole at the other. The operator is advifed to have by him feveral fuch wooden pieces of different length and thicknefs, that he may fhift them according as circumftances may require. We are informed, that the wood ufed for this purpofe fhould be rather of a foft kind than hard, as box wood and lignum vitæ are.

In order to throw the electric fluid with a director of this kind, a wire proceeding from the prime conductor is to be faftened to the wire wire of the director, which the operator muſt hold by the extremity of the glaſs handle, and manage ſo as to keep the wooden point at about one or two inches diſtant from the patient's body. Mr. Cavallo obſerves, that the electric fluid iſſuing from the wooden point has a power which is intermediate between that of the ſtream proceeding from a metal point, and the power of the ſparks; but yet it is in general the moſt efficacious method of electrization, and therefore no pains ſhould be ſpared in order to adminiſter it in the beſt poſſible manner.

Gentle as this method of electrifying may appear, our author aſſures us, that it will nevertheleſs be found too ſtrong for ſome perſons, eſpecially when uſed for open ſores upon delicate parts; in which caſes the wooden point muſt be removed, and the electric fluid ſimply thrown from the metal point of the director, which muſt now be kept at a greater diſtance than when the wooden piece was upon it. The electric fluid iſſuing out of this pointed wire of the director occaſions only a gentle wind upon the part towards which it is directed.—It might naturally be ſuſpected that ſo gentle, and nearly inſenſible a treatment, could hardly be of any efficacy; but the author affirms, that this mode of of electrization, viz. the throwing the fluid with a metal point, has often mitigated pains, and cured obstinate and dangerous diseases, which could not be removed by any other remedy. He informs us, that in general this treatment proved as efficacious to persons of delicate nervous constitutions as the other, or the throwing the fluid with a wooden point, does to those of ordinary constitutions. We are farther told, that in several cases, especially of open sores, the electric fluid issuing out of a wooden point has constantly increased the pain, and even enlarged the sore; whereas the fluid issuing out of the metal point has effectually diminished both.

The author observes, that the stream issuing out of a wooden point may be directed towards the eyes of the patient, without any apprehension of hurting him; but he allows there may be some cases, though he has seldom heard of any, in which this treatment may be thought to be too strong, and in such the metal point only may be used.

Amongst other methods of electrifying a diseased part of the body, Mr. Cavallo describes that of drawing sparks through flannel, which he tells us has proved singularly beneficial in cafes of palfy, rheumatifm, coldnefs of any particular part, &c. It is performed by placing the patient on the infulating ftool, fo that he may communicate with the prime conductor, and then a dry and warm flannel is fpread over the naked part that is to be electrified, and fparks are drawn through it by means of a director.

The author concludes this part of his work with fome general rules for practice. He begins by advifing the practitioner to employ the fmalleft force of electricity that is fufficient to remove or alleviate any diforder. He obferves, that in judging of cafes proper to be electrified, experience fhews, that in general all kinds of obftructions, whether of motion, of circulation, or of fecretion, are very often cured or relieved by electricity; but that it has feldom entirely removed difeafes of long ftanding, although it generally relieves the patient in fuch cafes. He cautions us not to give fhocks to pregnant women, and obferves, that in cafes of gathering tumours, the beft method is to draw the fluid by means of a wooden point, or if that proves painful, by means of a metal point. In palfies and rheumatifm, fmall fparks, and alfo very fmall fhocks are recommended. Mr. Cavallo allows, allows, that ſtronger ſhocks may be ſometimes, though ſeldom, adminiſtered for a violent tooth-ach, and for ſome internal ſpaſm of no long ſtanding. In caſes of muſcular contraction, he adviſes us to electrify not only thoſe muſcles which are ſuppoſed to be contracted, but likewiſe their antagoniſts.

He obſerves, that when the ſtream of electric fluid is thrown either with a wooden or metallic point, the length of the operation ſhould be from three to ten minutes; that when ſhocks are adminiſtered, their greateſt number ſhould not exceed a dozen or fourteen, except when they are to be given to the whole body in different directions; and that the number of ſparks, when they are uſed, may generally exceed the number of ſhocks mentioned above.

His laſt rule relates to the electrization of children upon the inſulating chair. He obſerves, that as it is difficult to let them ſtay without motion, the moſt convenient method is, to let another perſon ſit in the inſulating chair, and hold the child whilſt the operator is electrifying him.

In the third part of the work we find an eſtimate of the effects of electricity, applied as a remedy for various diſorders. We are told that that rheumatiſm, even of long ſtanding, is relieved, and generally quite cured, by only drawing the electric fluid with a wooden point from the part, or by drawing ſparks through flannel. The operation is to be continued for about four or five minutes, and repeated once or twice every day. In caſes of deafneſs, it is ſometimes neceſſary to ſend very ſmall ſhocks (for inſtance, of one thirtieth of an inch) from one ear to the other. The author has conſtantly obſerved, that whenever the ear is electrified, the diſcharge of the wax is conſiderably promoted.—The tooth-ach, occaſioned by cold, rheumatiſm, or inflammation, is generally relieved; but when the body of the tooth is affected, the diſeaſe is frequently increaſed by electricity.

Swellings in general, not excepting even white ſwellings, are ſaid to have been cured by drawing the electric fluid with a wooden point for three or four minutes every day. The ſame method, we are told, is equally efficacious in inflammations of every ſort. In ophthalmia, the eye is directed to be kept open, and care is to be taken not to bring the wooden point very near it, for fear of cauſing any ſpark. Sometimes it is ſufficient to throw the fluid with a

 metal metal point; for in these cases, too great an irritation should always be avoided. After throwing the fluid for half a minute, a short time is to be allowed to the patient to rest and wipe away his tears, which generally flow very copiously; the operation is then to be continued again for another half minute, and repeated in this manner four or five times a day.

Our author acknowledges, that the gutta serena, though it has sometimes been cured by, has often resisted electricity. The method he recommends in cases of this sort, is to draw the electric fluid with the wooden point for a short time, and then to send half a dozen very small shocks from the back part of the head to the forehead. He speaks of an opacity of the vitreous humour, that was perfectly cured by electricity.

In cases of fistula lachrymalis, electricity is said to have had the best effects. Palsies are seldom perfectly cured by it, especially when they are of long standing; but they are generally relieved to a certain degree. In these cases, we are advised to use the wooden point, or to draw sparks through flannel for about five minutes every day.

In

In ulcers or open fores, the gentleft electrization with a wooden or even a metal point is to be ufed, and only for three or four minutes a day. It feldom fails, we are told, to leffen the inflammation, and promote the healing of the fore.

Cutaneous eruptions, according to our author, have been fuccefsfully treated with electrization: but in fuch cafes it is to be obferved, that the wooden point muft be kept at leaft fix inches from the part; for if the irritation is too great, the eruption will fpread inftead of diminifhing. An immediate warmth about the part is a fure fign that the electrization is properly adminiftered.

Mr. Cavallo informs us, that the difeafe commonly called *St. Vitus's Dance*, has frequently been cured by drawing fparks and giving gentle fhocks; that fcrophulous tumours, when they are juft beginning, are generally cured by drawing the electric fluid from the part; and that in cancers, the pains are alleviated by the fame means. But he acknowledges, that he has feen only one cafe of cancer where the tumour was much reduced in fize by electricity; and indeed we fufpect, that the accounts given of cures in this deplorable difeafe, have been but too often exaggerated.

Our author fpeaks of the good effects of electricity in beginning abfceffes, in fciatica, and even in incipient pulmonary inflammation. In nervous head-aches, the electric fluid is directed to be thrown with a wooden point all round the head fucceffively.

Although electricity has never been of ufe in advanced dropfy, yet we are affured that it has often proved ferviceable in that difeafe when juft beginning. The author even recommends it in gout, which he fays has in various inftances been cured by it. He likewife fpeaks of it as an effectual cure for agues; and in fuppreffion of the menfes, extols it as a fpeedy and fuccefsful method of cure. In this latter cafe, fmall fhocks are to be fent through the pelvis, and fparks drawn through the cloaths.

In the venereal difeafe, electrization has been generally forbidden; having ufually increafed the pains and other fymptoms. Our author afcribes this prohibition to its having been improperly adminiftered; and contends, that a very gentle electrization has been found particularly beneficial in various cafes of venereal affection, even when the difeafe has been of long ftanding.

Mr. Cavallo concludes this part of his work with an account of ten cafes in which electricity was fuccefsfully adminiftered. The firft is a cafe of violent ophthalmia, communicated to him by Mr. Partington. The diforder had refifted a variety of remedies for upwards of two months, and was accompanied with excruciating pains. The pupil of the eye was fo nearly clofed, that fcarce any of it could be feen. After the patient had been electrified every day for a fortnight, the inflammation was quite fubfided; and at the end of five weeks, the pupil was completely dilated.

The two next cafes, one of an eryfipelas, and the other of a fiftula lachrymalis, are copied from Mr. Lovett's *Electricity rendered ufeful*; the fourth is the cafe of Mr. Fergufon, who was cured of a fore throat by electricity, and is taken from his *Introduction to electricity.* The fifth and fixth are cafes of fuppreffed menfes, from a pamphlet lately publifhed by Mr. Birch. Cafe the feventh contains Mr. Partington's account of a cure of mufcular contraction by electricity, firft printed in the *Phil. Tranf.* Vol. LXVIII. Cafe the eighth is extracted from

Vol.

Vol. LXIX. of the fame work, and contains Dr. A. Fothergill's account of a cure of the St. Vitus's dance by electricity. Cafe the ninth contains Dr. Hart's account of the bad effects of electricity in a paralytic affection, from the *Phil. Tranf.* Vol. XLVIII. and the tenth is a cafe of mufcular rigidity, fuccefsfully treated by Dr. Watfon, F. R. S.

In an appendix to the work our author relates a few experiments, made with a view to fhew the effects of the electric fluid upon the human body.

We have given a pretty full account of this work, becaufe we think the fubject of it interefting, and the author feems to have treated it in a judicious manner. We think it neceffary, however, to make fome allowance for Mr. Cavallo's zeal as an electrician. Not being a medical man, it feems likely that in his view of a favourite remedy he has overlooked the effects of other methods that were probably employed along with it in dropfy, gout, and a variety of other difeafes, which he confiders as being curable by electricity. We fincerely hope that his publication may be the means of drawing the attention of medical practitioners to this fubject, fo that we may be enabled fully to afcertain certain the degree of confidence electricity merits in the cure of difeafes.

