# Errata

**Published:** 1781-06

**Authors:** 


					-p. 260, 1. 231 for being curable read as having Been cured,?

				

